# RNA interference-mediated silencing of eukaryotic translation initiation factor 3, subunit B (*EIF3B*) gene expression inhibits proliferation of colon cancer cells

**DOI:** 10.1186/1477-7819-10-119

**Published:** 2012-06-26

**Authors:** Zheng Wang, Jinxian Chen, Jianhua Sun, Zhe Cui, Hui Wu

**Affiliations:** 1 Department of General Surgery, Renji Hospital, Shanghai Jiaotong University School of Medicine, 145 Shandong Middle Road, Shanghai, 200001, China

**Keywords:** Eukaryotic initiation factor, Colon cancer cell SW1116, Proliferation

## Abstract

**Background:**

A key factor underlying the control of the cellular growth, size and proliferation involves the regulation of the total protein synthesis. Most often, the initial stages of mRNA translation are rate limiting, which involves a group of eukaryotic translation initiation factors (*EIFs*). Research advances focused on the inhibition of their expression and activity hold the key to the initiation and progression of tumor and tumor prognosis.

**Method:**

We performed RNA interference (RNAi) with the lentivirus vector system to silence the *EIF3B* gene using the colon cancer cell strain SW1116. The negative control included the normal target cells infected with the negative control virus whereas the knockdown cells included the normal target cells transfected with the RNAi target virus. We tested the inhibition resulting from the decreased expression of *EIF3B* gene on the proliferation rate of SW1116 cells, including the cell cycle, apoptosis and clonability.

**Results:**

Compared with the negative control, the impact of *EIF3B* gene expression in SW1116 cells on the levels of mRNA and protein in the knockdown group, was significantly inhibited (*P* <0.01). Furthermore, the cell proliferation rate and clonability were also significantly inhibited (*P* <0.01). The apoptosis rate increased significantly (*P* <0.05). A significant decrease in the number of cells in the G1 phase (*P* <0.01) and significant increases in S (*P* <0.01) and G2 phases (*P* <0.05) were observed.

**Conclusions:**

The silencing of *EIF3B* gene expression inhibits the proliferation of colon cancer cells.

## Background

It is widely accepted that the uncontrolled proliferation of tumor cells depends on increased protein synthesis and ribosomal quantity [1,2]. Recent research suggests that ribosomal protein synthesis plays a direct role during the tumor initiation. A key factor underlying the control of the cellular growth, size and proliferation involves the regulation of the total protein synthesis. Most often, the initial stages of mRNA translation are rate limiting. The first step involves formation of an initiation complex of 43S pre-ribosome with subunits of 40s small ribosome, methionine tRNAi and a group of eukaryotic translation initiation factors (*EIFs*), such as *EIFs* 1 and 2. The next step entails binding of the initiation complex of 43S pre-ribosome with the 5′ end of mRNA and then with the eukaryotic translation initiation factor EIF4F. Other initiation factors that are involved in the initial translation include: the multisubunit *EIF3; EIF5A*, which is involved in protein and ribosome synthesis and stimulates the linkage of polypeptide chain and extension of translation; active *EIF6,* which codes for an insoluble protein and is found in the nucleus and in the cytoplasm, functioning as a translation initiation factor and preventing the association of the 40S and 60S ribosomal subunits. It binds to the fibronectin type III domains of integrin, beta 4 (*ITGB4*) and may help link *ITGB4* to the intermediate filament cytoskeleton; and so on. As all these translation factors play a critical role in protein synthesis, research advances focused on the inhibition of their expression and activity hold the key to the initiation and progression of tumor and tumor prognosis.

This research was carried out by constructing a lentiviral vector containing RNA interference expression cassettes of *EIF3B* gene and introducing it into the colon cancer cell strain SW1116 to analyze the effect of *EIF3B* gene silencing on the proliferation of colon cancer cells.

## Methods

### Materials

The cell strain SW1116 of colon cancer was provided by the Shanghai Institute of Digestive Disease. The dry powder of RPMI-1640 cell culture fluid was purchased from Gibco, Oklahoma USA. The restriction enzyme was offered by New England Biolabs Inc., Ipswich, MA, USA. The pGCSIL-GFP carrier was purchased from the Shanghai Genechem Co., Ltd., Shanghai, China.

### Reverse transcription polymerase chain reaction (RT-PCR) analysis of *EIF3B* gene expression in tumor cells

The total RNA was extracted under RNase-free conditions (carried out according to the operating manual of Trizol from Invitrogen Corp., Carlsbad, CA) with glyceraldehyde-3-phosphate dehydrogenase (GAPDH) as an internal reference. Primer design was as follows. \The *EIF3B* upstream sequence was: 5′-CGGTGCCTTAGCGTTTGTG-3′; and the downstream sequence: 5′-CGGTCCTTGTTGTTCTTCTGC-3′. The GAPDH upstream sequence was: 5′-TGACTTCAACAGCGACACCCA-3′; its downstream sequence: 5′-CACCCTGTTGCTGTAGCCAAA-3′. The Access RT-PCR kit was used to perform single-step reverse transcription and PCR amplification. An aliquot of 5 μl of amplified products was subjected to electrophoresis on 2% agarose gel. The gels were examined under the UV lamp.

### Construct design: lentiviral-mediated small interfering RNA delivery system

We targeted the gene of interest by designing small interfering RNAs (siRNAs) using the design software developed by Ambion Corp., Naugatuck, CT, USA to select the best parameters for the RNA interference target. We determined the effective target sequence: PSC-1: GGGAGAGAAATTCAAGCAAAT (*EIF3B* mRNA). We designed the DNA oligonucleotides of siRNA (by Shanghai Genechem Co., Ltd): PSCSI2749-1: 5′- CCGGGGGAGAGAAATTCAAGCAAATTTCAAGAGAATTTGCTTGAATTTCTCTCCCTTTTTG-3;

PSCSI2749-2: AATTCAAAAAGGGAGAGAAATTCAAGCAAATTCTCTTGAAATTTGCTTGAATTTCTCTCCC-3′.

After annealing, the double-stranded DNA was digested with AgeI and EcoRI to linearize the pGCSIL-GFP vector. We modified the double-stranded DNA after annealing and linked it with the pGCSIL-GFP vector following the double digestion. We used calcium chloride to prepare competent cells of *Escherichia coli* afresh and cultured it at 37°C for 16 hours. We used computer-aided high-throughput cloning of bacteria in liquid medium and sent it to the Shanghai Genechem Co., Ltd for sequencing.

### Preparation and grouping of cells

The SW1116 colon cancer cells were cultured in the RPMI-1640 culture solution with fetal calf serum (volume fraction 10%) and incubated with 5% CO_2_ at 37°C. The cells that remained in the logarithmic phase were divided into two groups: negative control, in which the normal target cells were infected with negative control virus, and knockdown, in which the normal target cells were infected with RNAi target virus.

### Real-time PCR and western blot to test knockdown efficiency

The SW1116 cells that grew well on the day prior to viral introduction were recovered. The cell suspension was incubated with 5% CO_2_ at 37°C. When the degree of cell fusion reached 30%, adequate viral load was introduced in different groups, to a multiplicity of infection (MOI) value of 100. Following incubation for three days, the expression level of GFP was observed under the fluorescence microscope. The culture was continued if the efficiency of infection exceeded 50%. After incubation for five days, the cells were collected; the mRNA expression of the gene of interest was analyzed using real-time PCR for RNA interference. The upstream primer sequence for *EIF3B* gene was 5′-GCCAAATAATCACCAACA-3′ while the downstream primer sequence was 5′-CTGGTTCTGAGCAGGTTC-3′.

The cell culture solution was aspirated and the cells washed twice in phosphate-buffered saline (PBS). Adequate amount of pre-cooled 2 × lysis buffer was added. After deplating, the cells were transferred to the tube and then lysed on ice for 10 to 15 minutes. The protein concentration was determined and adjusted to 2 μg/μl. Next, 2 × loading buffer was added to each sample and subsequently boiled for five to ten minutes. Forty micrograms of total protein was loaded into each well containing 10% SDS-polyacrylamide gel, subjected to electrophoresis at 30 mA for two minutes, and then transferred to a polyvinylidene fluoride (PVDF) membrane at 400 mA for two hours. The membrane was blocked with 5% milk in Tris-buffered saline (TBS) for one hour at room temperature. The primary antibody was added and incubated with the membrane for two hours at room temperature, and then washed three times with TBS/Tween 20. A secondary antibody was added to the membrane and incubated at room temperature with gentle agitation. Two hours later, the membrane was washed three times with TBS/Tween 20 for 10 minutes per wash. The bands were visualized using an enhanced chemiluminescence (ECL) kit followed by exposure to X-ray film.

### Cellomics to test inhibition of colon cancer proliferation following downregulation of the *EIF3B* gene

After trypsinization, the cell suspension was resuspended in complete medium and the density adjusted to 2 × 10^4^/mL. We used the blood counting chamber to count the cells, laying them at 2000 cells/well. Each group comprised three to five compound perforations. Each perforation was filled with 100 μl, and same quantity of cells. The cells were incubated with 5% CO_2_ and cultured at 37°C. Starting the next day, the plates were tested and read once a day with Cellomics for five days. After adjusting the input parameters of Cellomics ArrayScan (Thermo Fisher Scientific Inc, Waltham, MA, USA), the quantity of cells with green fluorescence were accurately calculated while scanning the perforations in the plates. The data were collected and analyzed to create a proliferation curve for the five days.

### Fluorescence-activated cell sorting (FACS) to assess inhibition of colon cancer cell cycle following *EIF3B* gene silencing

The cell culture supernatant was aspirated when the coverage rate for 6 cm dish cells in experimental group increased to 80%, ensuring that the cells did not enter the plateau phase. The cells were washed once with the D-Hank’s solution and subjected to trypsinization. The complete medium was removed and the cells were collected into a 5 ml centrifuge tube. We set three compound perforations in each group and performed timed cycle tests ensuring adequate number of cells for computerized analysis, with at least 1,000,000 each time. We used PBS (pH = 7.2 to 7.4) that was pre-cooled at 4°C to wash and precipitate cells once. The cells were collected after centrifugation at 1500 rpm for five minutes. The cells were fixed with 70% ethanol, which was pre-cooled at 4°C, for at least one hour. The stationary liquid was abandoned by centrifugation at 1500 rpm for five minutes. We used the PBS to wash and precipitate cells once. Adequate quantity of cell staining fluid (1 to 1.5 ml) was added for resuspension, based on the volume of cells, ensuring the pass rate of cells reached 200 to 350 cell/s for computer analysis. We used 300 mesh screen cloth to filter within the tube while streaming onto computer.

### Fluorescence-activated cell sorting (FACS) analysis of apoptosis inhibition following downregulation of the *EIF3B* gene

We used D-Hank’s solution to wash cells once after collecting the culture supernatant from each experimental group following transfer into the 5 ml centrifuge tube. We used pancreatic enzymes to digest the cells. The culture supernatants were then abandoned, and cells collected into one 5ml centrifuge tube, with three compound perforations in each group. The supernatants were aspirated after centrifugation at 1500 rpm for five minutes. We used PBS to wash and precipitate cells once, then collected cells after centrifugation at 1500 rpm for five minutes. A 1 × binding buffer was then used to wash and precipitate cells once, and centrifuge at 1500 rpm for five minutes, The collected cells were resuspended using 1 ml 1× staining buffer; the volume of staining buffer solution was determined according to the precipitation capacity of cells, adjusting the final density of cell suspension to 1 × 10^6^–1 × 10^7^ cell/ml). We took 100 μl cell suspension (1 × 10^5^–1 × 10^6^ cells) and added 5 μl annexin V-APC for dyeing. The mixture was placed in a dark place at room temperature for 10 to 15 min, and then transferred to the tube for streaming onto computer for further analysis.

### Inhibition of clonability of the cells following downregulation of the *EIF3B* gene

We digested cells that remained in the logarithmic phase in each experimental group, with pancreatic enzymes. The cells were resuspended in complete medium. Using a blood counting chamber, we inoculated the 96-well culture plate at the rate of 500 cells per perforation in each experimental group. We set three compound perforations in each experimental group, transferred the cells that were already inoculated into the incubator and they were cultured for three days or when the number of cells in most single clones exceeded five. During the process, the solution was changed and cells monitored every three days. Using Cellomics ArrayScan, we scanned and photographed the perforations, analyzed the number and size of clones within the perforations and the number of cells in each clone.

### Statistical methods

All data are presented as mean ± standard deviation ($\overset{￣}{x}\pm SD$). We used the statistical software SPSS12.0 to perform the relevant analysis. The significance level of statistics was set at *P* <0.05.

## Results

### Expression of *EIF3B* gene in SW1116 cells, preparation of RNA-interfering lentivirus vector and test of knockdown efficiency

**Figure 1:**
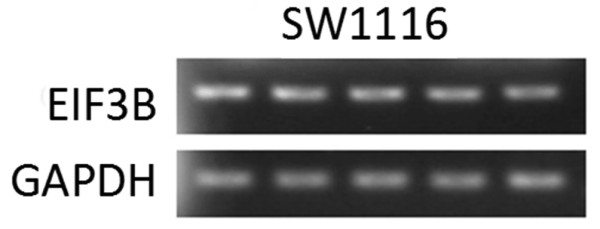
***EIF3B *mRNA expression in SW1116 cells.**

The results of the semi-quantitative PCR, which set GAPDH as an internal reference, showed that *EIF3B* gene was abundantly expressed in colon cancer cell SW1116 (Figure 1).

**Figure 2:**
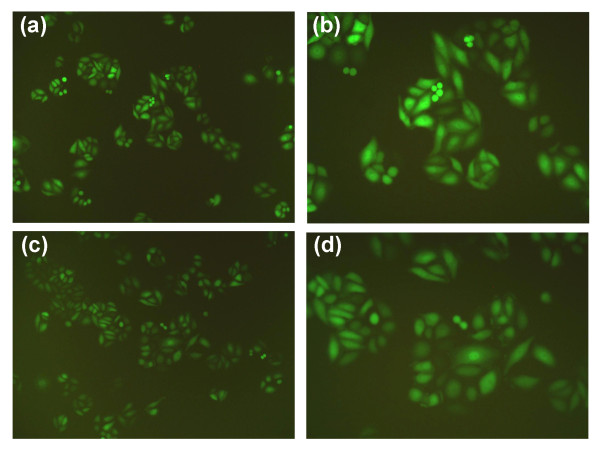
**GFP expression in SW1116 cells under fluorescence microscope. (a) ***EIF3B *-siRNA × 100. **(b) ***EIF3B *-siRNA × 200. **(c) ** Scr-siRNA × 100. **(d)** Scr-siRNA × 100.

**Figure 3:**
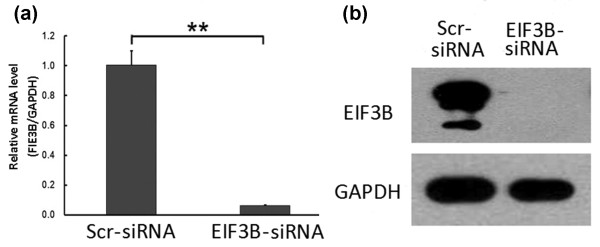
***EIF3B*gene expression in SW1116 cells of the knockdown group. (a) ** In the knockdown group, the mRNA expression of *EIF3B * gene in SW1116 cells decreases (**P  <0.01 *EIF3B *-siRNA versus Scr-siRNA). **(b) ** In the knockdown group, the protein expression of *EIF3B * gene in SW1116 cells decreases.

The length of PCR fragment in the positive clone that anneals with the fragment of vshRNA was 343 base pair. After transfection with siRNA lentivirus for three days, the GFP expression was observed under fluorescence microscope (Figure 2). After five days, it was found that the mRNA expression level of *EIF3B* gene in SW1116 cells of the knockdown group was inhibited, which was significantly different compared with the negative control group (*P* <0.01). The western blot results suggested inhibition of the protein level by the silenced *EIF3B* gene in SW1116 cells. The RNA-interfering lentivirus of the *EIF3B* gene construct effectively inhibited the expression of *EIF3B* gene and several targets following the gene silencing (Figure 3).

### Cellomics analysis of inhibition of the proliferation of colon cancer cells following downregulation of *EIF3B* gene

**Figure 4:**
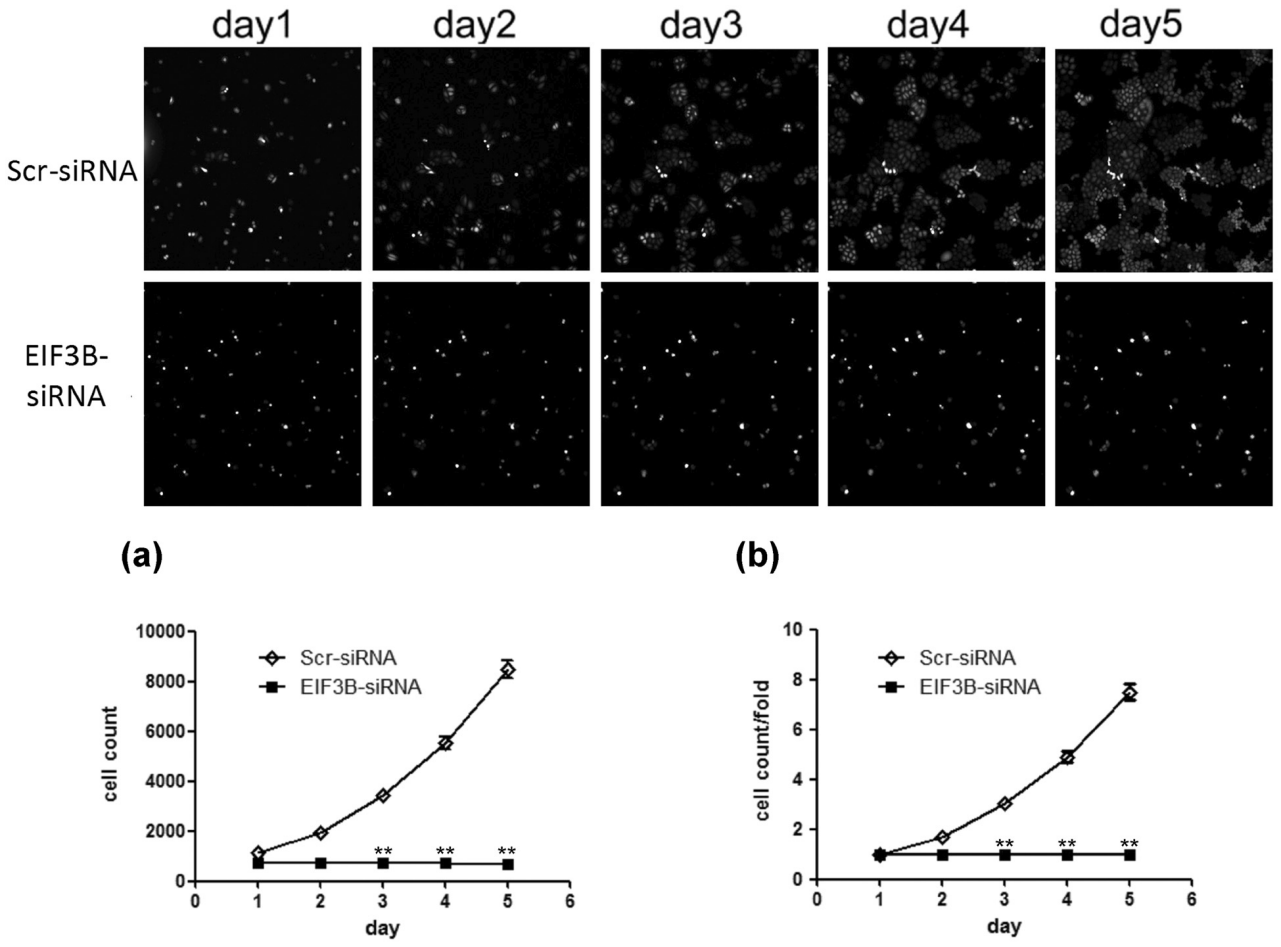
**Downregulation of*EIF3B*gene inhibits proliferation of SW1116 cells. (a) ** Proliferation volume of SW1116 cells in the knockdown group is inhibited together with the negative control group (**P <0.01 *EIF3B*-siRNA versus Scr-siRNA). **(b) ** Proliferation multiple of SW1116 cells in the knockdown group is inhibited together with the negative control group (**P <0.01 *EIF3B *-siRNA versus Scr-siRNA).

After transfection with siRNA lentivirus, the proliferation volume of several SW1116 cells in the knockdown group was inhibited together with the negative control from the third day (*P* <0.01). The results suggested that downregulation of *EIF3B* gene inhibited the proliferation of SW1116 cells (Figure 4).

### FACS analysis of cell cycle inhibition due to *EIF3B* gene silencing

**Figure 5:**
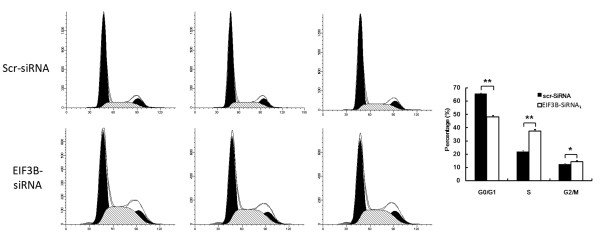
**SW1116 cell cycle changes in the knockdown group.** (**P <0.01 *EIF3B *-siRNA versus Scr-siRNA, *P <0.05 *EIF3B *-siRNA versus Scr-siRNA).

Following transfection with siRNA lentivirus, the SW1116 cells in G1 phase decreased significantly (*P* <0.01), while cells in S (*P* <0.01) and G2 phases increased significantly (*P* <0.05) in the knockdown group compared with the negative control group. It indicates that downregulation of *EIF3B* gene was apparently related to regular distribution of SW1116 cells (Figure 5).

### FACS analysis of apotopsis inhibition following *EIF3B* gene silencing

**Figure 6:**
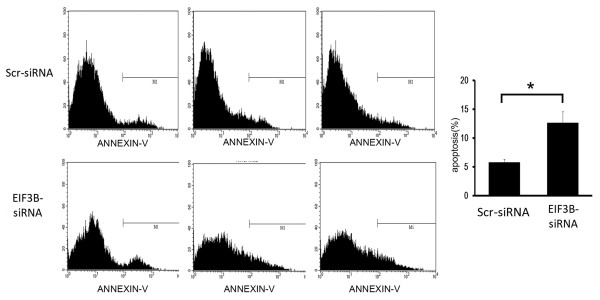
**Downregulation of*EIF3B*gene stimulates apoptosis of SW1116 cells (Peak diagram).** (*P <0.05 *EIF3B *-siRNA versus Scr-siRNA).

**Figure 7:**
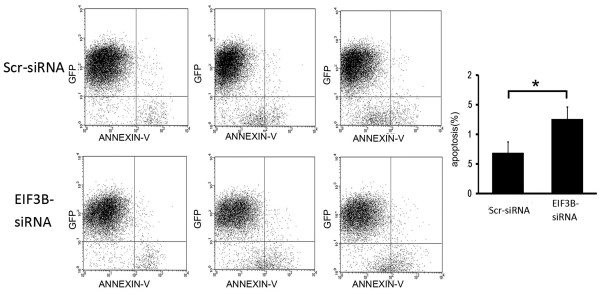
**Downregulation of*EIF3B*gene promotes apoptosis of SW1116 cells (Scatter diagram).** (*P <0.05 *EIF3B *-siRNA versus Scr-siRNA).

Transfection by siRNA lentivirus increased the volume of apoptosis significantly (*P* <0.05) in the knockdown group compared with the negative control group, suggesting that *EIF3B* gene silencing stimulated apoptosis of SW1116 cells (Figure 6) (Figure 7).

### Analysis of inhibition of clonability due to *EIF3B* gene silencing

**Figure 8:**
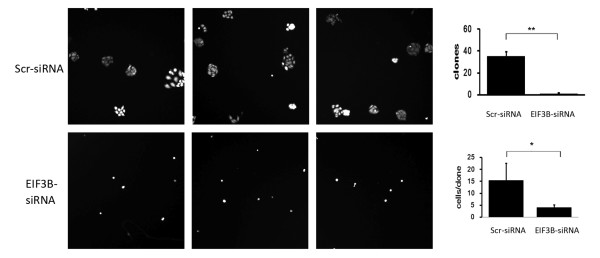
**Compared with the negative control group, the clonability of SW1116 cells in the knockdown group is significantly inhibited.** (*P <0.01 *EIF3B *-siRNA versus Scr-siRNA).

We noticed a decrease in the volume of SW1116 cell colonies in the knockdown group following interference by siRNA lentivirus. Furthermore, the volume of cells in the colony that had already formed decreased, which was significantly different from the negative control group (*P* <0.01). The results suggest that downregulation of *EIF3B* gene largely inhibited the clonability of SW1116 cells (Figure 8).

## Discussion

Recent research on mRNA transcription and protein synthesis in malignant tumor demonstrates the key role played by translational control in the tumorigenesis pathways. The mechanisms may entail the general control of protein synthesis and the selective translation of special mRNAs resulting in cancer cell survival, revascularization, mutation, invasion and metastasis. A variety of translational control mechanisms exist in tumors, among which the level of translational factors is critical. Since most translational control of mRNA occurs at the initial stage, it is extremely significant to understand the inhibition of expression and variations in activity of translational initiation factors and their impact on the growing tumor.

Eukaryotic initiation factors exist in several classes and series of subunits each. The *EIF2* includes α, β and γ subunits. When the *EIF2α* undergoes phosphorylation, it stops the recovery of GTP on *EIF2α* from *EIF2β*, thereby stopping the protein synthesis. Research suggests that mouse tumors were promoted by inhibiting the phosphorylation of *EIF2α*[3,4]. The *EIF5A* is a highly conserved translational initiation factor, which is necessary for cell proliferation. It occurs in two types in human cells: *EIF5A1*, which is the expressive type; and *EIF5A2*, which is the restricted type. The synthesis and increase of protein and ribosome in the two *EIF5As* are associated with the polypeptide chain linkages, but also are important steps in translational extension [5]. In the transplantation model of heterogeneous tumor within mouse, the overexpression of *EIF5A2* stimulated tumor growth. The *EIF6* is a multifunctional translational factor. Its main function is related to the creation of ribosome within nucleus [6]. Furthermore, it modified the activity of ribosomal subunits in cytoplasm. The activity of *EIF6* is probably related to its location within the cell. Research suggests that decreasing the *EIF6* level in cytoplasm inhibited the development of tumor while increasing the level of nucleolus-stimulated tumor formation [7]. The *EIF4* is an important translational factor stimulating the development of tumor. It includes: the *EIF4E*, which is the cap-conjugated protein; the *EIF4A*, which is an adenosine triphosphate (ATP)-dependent RNA helicase; RNA-conjugated protein, similar to *EIF4BEIF4G* and so on [8]. In the transgenic mouse model, the overexpression of *EIF4E* caused B cell lymphoma, lung cancer, liver cancer and angiosarcoma [9,10]. The *EIF4A* is overexpressed in cell lines of melanoma, some primary melanomas and primary hepatocellular carcinoma [11]. In some tumor-bearing animal models, depleting the *EIF4A* from the *EIF4F* complex pharmacologically inhibited the initial synthesis of protein and increased the drug sensitivity of tumor. The *EIF4G1* is overexpressed in scaly lung cancer [12,13].

The mammalian *EIF3* complex contains 10 to 13 proteins. Its main function is to adjust the interaction between ribosome and mRNA, often the initial process of protein synthesis. The *EIF3* complex is related to the 40S ribosome. It is also useful in the addition of *EIF1EIF1AEIF2*, GTP, methionine tRNAi and *EIF5* to the 43S ribosomal complex. The linkage of *EIF3* complex and *EIF4F* stimulates the mRNA addition to 43S ribosomal complex. In addition, the disassembly and recycling of terminal ribosome also require *EIF3*. At the same time, it prevents the early association between subunits of 40S ribosome and 60S ribosome before translation [14-16]. Because of its key role in the process of protein synthesis, the *EIF3* has a variety of functions in malignant tumors. EIF3 consists of many subunits, which include *EIF3aEIF3BEIF3cEIF3i* and so forth, each with its own function. Several of these subunits form the core complex, which is necessary for the protein synthesis, while other subunits are useful in adjusting the function of *EIF3* complex or initiating the internal interactions between EIF3 and special mRNA [17]. EIF3a is abundantly expressed in many tumors within the human body. Antisense RNA decreased its expression *in vitro* and reversed the malignant phenotype. The overexpression of EIF3c caused many kinds of tumors within the human body, via excessive protein translation. It may help with protein synthesis, protein binding and restrict the inhibitory factors of tumor. *EIF3H* usually is overexpressed in prostate cancer and breast cancer. In small cell lung cancer, the overexpression of *EIF3H* indicates a positive therapeutic response to Iressa therapy. The *EIF3F* and *EIF3E* have characteristics similar to tumor inhibitory factors, which inhibit the cell proliferation and promote apoptosis. *EIF3F* lacks expression in melanoma, unlike in benign pathological change of skin, such as mole [18-20]. In breast cancer and lung cancer within the human body, the expression of *EIF3E* decreases [21-23]. *EIF3B* is an important part of the *EIF3* complex, involved in tumor formation. The overexpression of *EIF3AEIF3BEIF3C* and *EIF3I* subunits stimulates the NIH3T3 cell total protein synthesis and promotes the translational function of special RNAs such as cycin D, Myc, ornithine decarboxylase (ODC) and the growth factor of collagenous fiber alpha [24].

## Conclusion

In our study, using the RNA interference with lentivirus vector containing the *EIF3B* gene, we knocked down the expression of *EIF3B* gene in the colon cancer cell strain SW1116. After successful downregulation of *EIF3B* mRNA and protein expression, the proliferation rate and clonability of SW1116 cells were also inhibited significantly. The apoptosis increased significantly while the cell cycle was influenced. Downregulation of *EIF3B* gene expression inhibited the proliferation of colon cancer cells.

Changes in protein synthesis and translational control play a critical role in the complex mechanism underlying tumor formation. They include changes in protein synthesis and selective translational control of mRNA, tumor vessel formation, inhibition of tumor cell apoptosis, increased proliferation of tumor cells, and interaction between tumor cells and microenvironment. Research into the underlying mechanisms may have implications for therapeutic outcomes.

## Abbreviations

EIFs, eukaryotic translation initiation factors; RNAi, RNA interference; ITGB4, integrin beta 4; GAPDH, glyceraldehyde-3-phosphate dehydrogenase; siRNAs, small interfering RNAs; MOI, multiplicity of infection; PBS, phosphate-buffered saline; PVDF, polyvinylidene fluoride; TBS, Tris-buffered saline; ECL, enhanced chemiluminescence; FACS, Fluorescence-activated cell sorting.

## Competing interests

The authors declare they have no competing interests.

## Authors’ contributions

ZW carried out the molecular genetic studies, participated in the sequence alignment and drafted the manuscript. JHS carried out cell separation and culture. ZC participated in the design of the study and performed the statistical analysis. HW conceived the study, participated in its design and coordination, and helped to draft the manuscript. JXC is the guarantor of the integrity of the entire study. All authors read and approved the final manuscript.
